# Improving the diagnosis of urinary tract infections by the use of enriched media and a 48-hour incubation period

**DOI:** 10.1099/jmm.0.001846

**Published:** 2024-06-27

**Authors:** Carla Benjumea, Ferran Navarro, Carles Alonso-Tarrés

**Affiliations:** 1 Microbiology Department Laboratory and Infection Control, Fundació Puigvert, Barcelona, Spain; 2 Departament de Genètica i Microbiologia, Universitat Autònoma de Barcelona, Cerdanyola del Vallès, Barcelona, Spain; 3 Microbiology Department, Hospital de la Santa Creu i Sant Pau, Barcelona, Spain; 4 Departament de Genètica i de Microbiologia de la Universitat Autònoma de Barcelona, Institut d’Investigació Biomèdica de Sant Pau (IIB Sant Pau), Barcelona, Spain

**Keywords:** blood agar, comparison, culture, incubation, isolates, microbiology, nephrology, urine, urine culture, urology, UTI Brilliance Clarity agar

## Abstract

**Introduction.** The absence of a gold-standard methodology for the microbiological diagnosis of urinary tract infections (UTI) has led to insufficient standardization of criteria for the interpretation of results and processing methods, particularly incubation time and culture media.

**Hypothesis.** 48-hour incubation time period and use of blood agar enhances the sensitivity of microorganisms isolated significantly.

**Aim.** To determine the sensitivity of blood agar and Brilliance UTI chromogenic agar, incubating for different periods (24–48 hours), for the detection of positive urine cultures.

**Methodoloy.** Comparisons were made between all possible combinations of media and incubation times. As the gold-standard reference, we used the routine methodology of our laboratory, which involves prior screening with available clinical data, flow cytometry, sediment analysis and/or Gram staining. Screened samples were then cultured on blood agar and chromogenic agar and incubated for 48 hours. Also, based on the results of Gram staining, additional media were added in selected cases.

**Results.** The most significant difference was found between chromogenic agar incubated for 24 hours and blood agar incubated for 48 hours, with the latter method allowing the recovery of 10.14 % more microorganisms (*P* < 0.0001). Furthermore, the value of performing Gram staining to guide processing was demonstrated, as it avoided the loss of at least 5.14 % of isolates.

**Conclusions.** At least in urological and nephrological patients it is essential to include enriched culture media (blood agar) or to extend the incubation times due to the improvement of the diagnostic sensitivity of urine cultures. Gram staining also can help detect the presence of fastidious microorganisms or mixed infections, indicating whether rich and/or selective media should be included to enhance the diagnostic sensitivity of cultures. If this methodology is not followed, it should be noted that besides fastidious species, fastidious strains of *Escherichia coli, Proteus mirabilis, Pseudomonas aerugniosa* and *Stenotrophomonas maltophilia* will also be missed.

**Impact Statement**

The data provided here indicate that, depending on the patient population, diagnostic laboratories could improve diagnosis of urinary tract infections by using enriched culture media and longer incubation times. The reported results are particularly relevant for institutions that handle a large number of urological patients, as in the present study.

## Introduction

The diagnosis of urinary tract infections (UTIs) is primarily clinical and based on the patient’s signs and symptoms. Although the culture methods currently used are not uniform across laboratories, urine culture is the gold standard for laboratory diagnosis of UTIs. Also, methods are available that help to confirm a suspected diagnosis, including test strip screening, examination of urinary sediment and Gram staining.

Currently, there is no universally accepted gold standard for laboratory diagnosis of UTI, and a range of recommendations and general guidelines are adopted according to the specific needs of each laboratory. These recommendations advise performing urine cultures on cysteine–lactose–electrolyte-deficient agar or chromogenic agar (CRA), as well as on blood agar (BA), both types of culture being incubated for 24 hours. Optionally, a prolonged 48-hour incubation period and/or the use of other media cultures are suggested [1,5].

The result is a plethora of working methodologies with significant variations. At one end of the spectrum, some laboratories only use urine culture for UTI diagnosis, while at the other end, test strip screening, cyto-bacteriological examination, Gram staining and urine culture are performed. One of the most used methods involves test strip analysis, and, if the results indicate urinary tract pathology (presence of leucocyturia, haematuria and/or nitrites), confirmation by urine culture is needed. It is also common to include the examination of urinary sediment (manual or automated) to determine the presence of bacteria, leucocyturia and haematuria [4,6,10]. Although guidelines recommend a longer BA culture of 48 hours [3], many laboratories still use a period of 18–24 hours [11,14]. In fact, in some published studies, the incubation time of cultures is not even mentioned [10].

Previous comparative studies of culture media and incubation times for UTI diagnosis [15,20] all share limitations regarding the evaluation of urine cultures. For example, cultures with counts below 10^4^ c.f.u./ml are regarded as negative, which can lead to potential infection-causing organisms being missed and introduce bias into the study results. Similarly, failing to consider the sample collection method or patient symptoms makes it difficult to identify whether a microorganism is a contaminant or a causative agent of infection, again providing misleading results. Normally, saprophytic microorganisms that colonize the skin are regarded as contaminants, but in certain circumstances, they should be treated as opportunistic pathogens, as in the case of coagulase-negative staphylococci [21].

Due to the absence of a gold standard and the variability of available methodologies, the aim of this study was to assess and quantify the performance of BA incubated in an atmosphere enriched with 7 % CO_2_ for 48 hours compared to other methods (CRA incubated for 24 or 48 hours and BA incubated for 24 hours) and to determine the clinical and statistical significance in a nephrological and urological patient population.

## Methods

### Sample processing

The study was conducted at the monographic Puigvert Foundation hospital, which specializes in nephrology, urology and andrology. All urine samples were obtained at this hospital from patients who were hospitalized, being treated in the emergency room or attending outpatient consultations. The samples were transported to the microbiology laboratory within 2 hours and processed immediately.

The diagnostic algorithm for UTIs at the Puigvert Foundation hospital, taken as a reference method (referred to henceforth as the PF method), involves a test strip screening of all samples using automated urine analysers UC-3500 (Sysmex Europe) or Cobas u411 (Roche Diagnostics). For clean-catch urine, the following test strip parameters were regarded as altered: positive for leucocyte esterase (≥10 /µl) and nitrites, haematuria ≥10 /µl, pH higher than 8 or proteins exceeding 1.50 g/l. If an altered value was obtained or if the urine appeared turbid but without altered values, the samples were analysed further using flow cytometry (UF-4000 or UF-5000 Sysmex Europe).

For non-clean-catch urine samples, i.e. those obtained through bladder catheterization (including vesical, nephrostomy, urostomy and cystostomy catheters), as well as urine from patients under 6 years of age, neutropenic patients or pregnant women, a culture was performed regardless of whether the test strip and flow cytometry showed altered values or not. In samples with a large amount of proteins or cells or the presence of cylinders, crystals or yeast, the analysis was complemented with a manual optical microscopic examination of urine sediment.

Gram staining was performed on urine samples with a bacterial count greater than 1000 /µl by flow cytometry. Samples observed to be contaminated with vaginal microbiota and containing squamous epithelial cells (>4.5 per field at 400× magnification) were regarded as contaminated.

Finally, clean-catch urine samples with more than 10 leucocytes per field (×400) and not contaminated according to microscopic criteria were cultured. All samples positive in the strip test screening were seeded on Columbia BA with 5 % sheep blood (Becton Dickinson, Germany) and Oxoid Brilliance UTI Clarity agar (Thermofisher, United Kingdom). In some cases, additional media such as Columbia CNA agar with 5 % Sheep Blood, Improved II (CNA-I) (Becton Dickinson, Germany) and Candida Brilliance agar (CandidaBrilliance agar, Thermofisher, United Kingdom) were added, depending on the microorganisms observed in Gram staining.

All urine samples were plated using a semi-quantitative streak method with a calibrated 10 µl bacteriological loop. The CRA plates were incubated aerobically at 35 °C, and BA plates were incubated in an atmosphere enriched with 7 % CO_2_ at 35 °C.

### Data collection and interpretation of results

An initial reading of both plates was conducted at 18–24 hours of incubation, followed by a second reading at 48 hours. Samples that could not be read at 24 and 48 hours because of weekends or holidays were excluded from the study. A culture was considered negative only when no growth was observed. When mild growth was apparent – insufficient to work with but visible – it was counted as positive at the corresponding incubation time.

The interpretation of urine cultures was primarily based on CUMITECH (2C) criteria, including urinalysis data (assessment of leucocyturia, collection method, clinical syndrome and the number of isolates) [1,3, 22].

The different culture methods were compared according to the number of isolated species (see Tables1  2) and positive urine cultures (see Tables S1 and S2, available in the online Supplementary Material). The two variables in the comparison were the culture medium (BA or CRA) and the incubation time (24 or 48 hours). The gold-standard PF method was CRA + BA incubated for 48 hours plus additional selective plates according to the Gram-staining results. As the reference method includes the methods under study in its intermediate steps (BA and CRA read at 24 hours) and final interpretation, the specificity of the different methods was not determined, only their sensitivity.

**Table 1.** Microorganisms isolated using the PF method. Number of lost isolates and the percentage they represent within each medium at each incubation time

Microorganism	No. of isolates in PF method	No. of lost isolates in CRA 24 hours	No. of lost isolates in CRA 48 hours	No. of lost isolates in BA 24 hours	No. of lost isolates in BA 48 hours
*Escherichia coli*	109	2	1	0	0
*Klebsiella pneumoniae*	53	1	1	0	0
*Pseudomonas aeruginosa*	27	3	0	0	0
*Enterococcus faecalis*	27	0	0	0	0
*Proteus mirabilis*	24	2	0	1	1
*Staphylococcus aureus*	12	4	3	2	2
*Enterobacter cloacae*	6	0	0	0	0
*Klebsiella oxytoca*	6	0	0	0	0
*Staphylococcus epidermidis*	5	3	0	0	0
Streptococci from ‘*viridans’* group	5	3	3	1	0
*Streptococcus anginosus*	4	4	3	0	0
*Candida parapsilosis*	4	3	1	0	0
*Citrobacter koseri*	4	0	0	0	0
*Serratia marcescens*	4	0	0	0	0
*Actinotignum schaalii*	3	3	3	2	1
*Corynebacterium urealyticum*	3	3	3	0	0
*Enterobacter aerogenes*	3	0	0	0	0
*Enterococcus faecium*	3	0	0	0	0
*Staphylococcus hominis*	3	0	0	0	0
*Streptococcus bovis*	2	1	1	1	1
*Staphylococcus haemolyticus*	2	0	0	0	0
*Streptococcus agalactiae*	2	0	0	0	0
*Proteus vulgaris*	2	0	0	0	0
*Citrobacter freundii*	2	0	0	0	0
*Morganella morganii*	2	0	0	0	0
*Candida albicans*	2	0	0	0	0
*Stenotrophomonas maltophilia*	1	1	0	1	0
*Candida glabrata*	1	1	1	1	0
*Streptococcus sanguinis*	1	1	0	0	0
*Aerococcus viridans*	1	0	0	0	0
Coagulase-negative staphylococci	1	0	0	0	0
*Proteus penneri*	1	0	0	0	0
*Alcaligenes faecalis*	1	0	0	0	0
*Enterobacter cancerogenus*	1	0	0	0	0
*Acinetobacter baumanii*	1	0	0	0	0
*Aerococcus* sp*.*	1	0	0	0	0
*Serratia* sp.	1	0	0	0	0
*Candida tropicalis*	1	0	0	0	0
Total	331	35 (11 %)	20 (6 %)	9 (3 %)	5 (2 %)

The percentage of lost microorganisms in each case is relative to the total isolates of the same genus/species.

A total of 331 isolates were obtained from 266 positive cultures.

**Table 2.** Comparison of the culture methods according to the number of lost microorganisms

Method 1 vs method 2	No. of MOO lost in method 1 compared to method 2	*P* (McNemar)	Percentage of MOO lost in method 1 out of total MOO (*N* = 331)	Confidence interval (95 %)*Z*-score	Percentage increase of method 2 compared to method 1	Confidence interval (95 %)*Z*-score
CRA 24 hours – BA 48 hours	30	<0.0001	9.06	6.19–12.68	10.14	6.95–14.16
CRA 24 hours – PF method	35	<0.0001	10.57	7.47–14.39	11.82	8.37–16.05
BA 24 hours – BA 48 hours	4	0.125	1.21	0.33–3.07	1.24	0.34–3.15
BA 24 hours – PF method	9	0.0039	2.72	1.25–5.10	2.80	1.29–5.25
BA 48 hours – PF method	5	0.0625	1.51	0.49–3.49	1.53	0.50–3.54
CRA 48 hours – BA 48 hours	15	0.0005	4.53	2.56–7.36	4.82	2.72–7.83
CRA 48 hours – PF method	20	<0.0001	6.04	3.73–9.18	6.43	3.97–9.76
CRA 24 hours – BA 24 hours	26	<0.0001	7.85	5.19–11.29	8.78	5.82–12.60
CRA 24 hours – CRA 48 hours	15	0.0001	4.53	2.56–7.36	5.07	2.87–8.22

Method 1 is named on the left side of the hyphen and method 2 on the right side.

Statistical calculations: *P*-value for the difference in proportions of isolates obtained with various methods and confidence intervals for the proportions of lost isolates. Calculation of the increase in percentage of isolates obtained by method 2 compared to method 1.

BAColumbia based blood agarCRAchromogenic agarMOOmicroorganisms

Furthermore, the number of UTIs diagnosed with each method was calculated. When the urine culture was negative by all methods, the UTI was regarded as microbiologically undiagnosed.

### Definitions

**Urinary tract infections.** Cases with compatible urinary symptoms, pathological sediment and a positive culture were considered UTIs, including cystitis, pyelonephritis and prostatitis.

**Asymptomatic bacteriuria**. Cases without urinary symptoms and a positive culture were considered asymptomatic bacteriuria.

### Statistical methods

In previous studies by our group (data not published), it was found that the percentage of fastidious microorganisms susceptible to being isolated only on BA was around 8 %–9 %. Based on these percentages, the sample size was calculated using a *χ*^2^ test for comparing two proportions, yielding a minimum of 186 samples. To increase the range of fastidious species recovered, it was decided to triple the sample size. Finally, a total of 428 samples were evaluated.

The statistical significance of differences in various comparisons was calculated using the McNemar exact test. Cultures were compared to calculate the sensitivity of each method. For all calculations, MedCalc software version 20.115 was used.

### Ethics committee

The study was conducted according to the Declaration of Helsinki and approved by the Ethics Committees of Puigvert Foundation (02 November 2023). The study had no impact on normal diagnostic and therapeutic procedures. Clinical information was obtained from electronic patient records, and the results were pseudonymized.

## Results

Out of all the urine cultures (*N* = 428), 99 were negative (no growth), and 63 were contaminated, resulting in a total of 162 negative readings according to the final clinical-microbiological assessment. Regarding the isolated microorganisms, a total of 331 strains were obtained in the study, either in a mixed or pure culture.

The analyses were divided into two groups, resulting in two types of results. The first group (Table 1) included all isolated microorganisms (*n* = 331), and the second group (Table S1) constituted the positive cultures (*n* = 266).

These *Escherichia coli* isolates not detected in CRA at any incubation time in ambient air were found to be fastidious bacteria that would only grow on a blood-based medium in CO_2_-enriched atmosphere, which is not frequent in this species. In one case, *Klebsiella pneumoniae* did not grow in CRA, as the sample was collected after the patient received antibiotics. Thus, due to the low quantity of microorganisms in the bladder, only one c.f.u. was observed in the final BA plate subculture.

One *Staphylococcus aureus* isolate was not detected in CRA at either 24 or 48 hours but grew in BA. Two other *S. aureus* isolates were not visualized in BA at either incubation time due to the overgrowth of other microorganisms; both were detected using the PF method, in which a CNA-I agar plate was added after the observation of gram-positive cocci in the Gram stain.

In Table 2, the different methods are compared according to the number and percentage of lost microorganisms.

Four out of the five lost microorganisms in BA at 48 hours compared to the PF method were strains isolated on CNA-I after the overgrowth of other microorganisms, such as *E. coli, Proteus mirabilis* and *Pseudomonas aeruginosa*, which prevented their visualization in BA. The isolates lost for this reason in BA and CRA at either incubation time were one *Streptococcus bovis*, two *S. aureus* and one *Actinotignum schaalii.*

The other lost microorganism in BA at 48 hours was *P. mirabilis*, which was isolated only in CRA. This was due to antibiotic administration to the patient before sample collection and because by chance the single c.f.u. of the bacteria failed to grow on BA. The same occurred with a *K. pneumoniae* isolate, which was detected only in CRA but not in BA due to the antibiotic in the sample.

Regarding the diagnoses, a total of 104 UTIs were confirmed by the PF method, some of which were missed by the other methods. Eleven UTI diagnoses were missed in CRA at 24 hours (10.58 %), three in CRA at 48 hours (2.88 %), two in BA at 24 hours, (1.92 %) and, finally, one in BA at 48 hours (0.96 %).

Although it was not the main focus of the study, we also determined whether the information obtained with Gram staining could facilitate the recovery of fastidious microorganisms by incorporation of enriched culture media. This method allowed 17 more cases (5.14 % of the total lost isolates) to be recovered (*P*-value <0.0001) compared to CRA 24 hours, resulting in the loss of only 13 cases. Thus, by adding enriched media according to Gram stain results, the percentage of lost isolates was reduced from 9.06 % to 3.93 %.

Finally, with the data obtained from the culture analyses, the sensitivity of each culture media at each incubation time was calculated, yielding the following results: CRA at 24 hours: 91.01 % (CI ±3.43), CRA at 48 hours: 96.63 % (CI ±2.16), BA at 24 hours: 98.13 % (CI ±1.63) and BA at 48 hours: 99.62 % (CI ±0.74).

## Discussion

Each clinical microbiology laboratory chooses to follow a UTI diagnostic procedure based on the needs and characteristics of the treated population, as reflected in various guidelines [1,2, 4, 6]. As the published information about the use of these procedures is scarce and out of date, we decided to assess the utility of a 48-hour BA culture in a 7 % CO_2_-enriched atmosphere compared to a 24-hour CRA culture in an aerobic atmosphere to determine the best method for a hospital attending predominantly urological and nephrological patients.

In this study, we found that using BA incubated for 48 hours increased the isolation of microorganisms causing some type of UTI or asymptomatic bacteriuria by 10.14 % (*P* ≤ 0.0001) and increased positive monomicrobial cultures by 9.39 % (*P* ≤ 0.0001, Table S1). In the former, each isolation was analysed individually, providing data on potentially lost microorganisms, whether part of a mixed or pure culture, whereas in the latter, only monomicrobial cultures were considered. The limitation of mixed cultures is that a single microorganism responsible for infection or colonization can be lost.

Furthermore, according to the interpretation criteria followed in this study, positive samples did not only include symptomatic UTIs but also asymptomatic bacteriuria (>100 000 c.f.u. ml^−1^) or other diagnoses (acute urinary retention, catheter obstruction, prostatitis, etc.). In some instances, lower counts (<100 000 c.f.u. ml^−1^) of the isolated microorganism were considered significant according to the guidelines [2,3, 23].

After performing various comparisons, it was evident that in most cases, culture in BA for 48 hours was significantly better than any of the other studied methods, in contrast with the results of previous studies [15,17, 19, 20]. As expected, the most significant difference was observed when comparing CRA 24-hour vs BA 48-hour cultures. In the former, almost all microorganisms regarded as fastidious bacteria were lost, such as viridans group streptococci, *Streptococcus anginosus*, *A. schaalii*, *Corynebacterium urealyticum* and *Streptococcus sanguinis*. Only one fastidious microorganism was isolated in this chromogenic medium (*S. bovis*). Moreover, it should be emphasized that microorganisms not considered fastidious, such as *E. coli, K pneumoniae, P. aeruginosa, P. mirabilis, S. aureus, Staphylococcus epidermidis* and *Stenotrophomonas maltophilia*, were also lost in CRA. These results reinforce the findings of Murray P. *et al.* and Joho K. *et al.*, who were among the first to report the existence of fastidious enterobacteria in urine. Therefore, finding the aforementioned fastidious microorganisms that would not grow in CRA is unlikely but possible [17,20, 24].

All lost *P. aeruginosa* isolates (*n* = 3) in CRA at 24 hours were recovered at 48 hours, whereas in BA, they were detected at 24 hours. *S. maltophilia* required 48 hours to grow in both types of culture media. It should be noted that only plates without any growth at 24 hours were considered negative. Similarly, within the group of enterobacteria, a lost *P. mirabilis* in CRA at 24 hours was recovered at 48 hours in the same medium. This finding was also observed but not considered important, despite statistical significance, in studies by Joho K. *et al.* and Cavagnolo R., as well as those of Murray P. *et al.* [17,19, 20, 25, 26].

Among gram-positive bacteria, two *S. aureus* failed to grow in CRA at 24 hours, only one of which was detected at 48 hours in the same medium. This finding aligns with the results of Joho K. *et al.*, who also reported a case of *S. aureus* not growing at 24 hours of incubation. The underlying cause of this has not been established.

Overall, incubating the CRA for 48 hours reduced the lost isolates from 10.6 % to 6.0 % (Table 1), i.e. they were halved. Those not recovered by extending the incubation time were fastidious microorganisms whose recovery requires a medium with blood. Fewer microorganisms were also lost by incubating BA for an additional 24 hours. Although the difference was not statistically significant, an incomplete or incorrect diagnosis can have important consequences for the patient’s health. A noteworthy example was the culture lost at 24 hours due to the absence of any growth of *S. maltophilia*, which is a multidrug-resistant microorganism that requires patient isolation in hospital.

Regardless of the culture media used or incubation time, some isolates were detected only by the PF method due to the use of Gram staining. This occurred in two cases where staphylococci colonies were not visualized in CRA at 24 hours or BA at 48 hours due to the overgrowth of other microorganisms. Following the PF algorithm, the staphylococci were detected in Gram stains, and a selective medium for Gram-positive bacteria (CNA-I agar with aztreonam) was added.

It should also be noted that if the sample has a low inoculum, as occurred in a case of *P. mirabilis* and *K. pneumoniae*, the cultures may only be positive by chance, regardless of the media/incubation times used.

Finally, it was studied whether the microorganisms lost in each method were causative agents of a UTI (cystitis, pyelonephritis or prostatitis). It was determined that they were responsible for a similar percentage of UTIs as in the overall population studied, without a statistically significant difference.

## Limitations

The study was conducted at a hospital specialized in nephrology, urology and andrology. Therefore, the results could at least be extrapolated to similar patient populations of other healthcare centres.

As the study was done following the routine methodology of the microbiology laboratory at the Puigvert Foundation, Gram stains were only performed on samples with more than 10^6^ c.f.u. ml^−1^ of bacteria observed through flow cytometry, and all cultured urines underwent a prior screening.

In this study we did not determine whether the lack of growth on CRA was due to the fastidious nature of the microorganism or if bacterial growth was inhibited by the interaction of some element of the urine sample with this culture medium. To do so, a subculture of the fastidious isolate on BA and on CRA should have been performed. Nevertheless, these discrepant isolates correspond to microorganisms widely documented as having difficult growth in chromogenic or unenriched media.

## Conclusions

In laboratories receiving samples from urological and nephrological patients, it is essential to include enriched culture media such as BA in the diagnostic algorithm to increase the number of microbiological diagnoses. The applicability of these findings in the general population should be studied in future research.

Extending the incubation times improves the diagnostic sensitivity of cultures, especially when using chromogenic media (96.63 %). This improvement is observed to a lesser extent when enriched media are used, as they already exhibit a very high performance at 24 hours (98.13 %).

Gram staining can help detect the presence of fastidious microorganisms or mixed infections, indicating whether rich and/or selective media should be included to enhance the diagnostic sensitivity of cultures (5.14 %, *P* < 0.0001). If this methodology is not followed, it should be noted that besides fastidious species, fastidious strains of *E. coli, P. mirabilis, P. aeruginosa* and *S. maltophilia* will also be missed.

## supplementary material

10.1099/jmm.0.001846 Uncited Table S1.

## Abbreviations

BA: Blood Agar
CFU: Colony Forming Unit
CLED: Cysteine lactose electrolyte deficient
CNA: Colistin, Nalidixic Acid agar
CRA: Chromogenic Agar
MOO: Microorganism
PF: Puigvert Foundation
UTI: Urinary Tract Infection
