# Supplementary material for: Improving the diagnosis of urinary tract infections by the use of enriched media and a 48-hour incubation period
Source: J Med Microbiol. 2024 Jun 27;73(6):001846. doi: 10.1099/jmm.0.001846 (PMC11261898; doi:10.1099/jmm.0.001846)
Supplement: Uncited Table S1. [file jmm-73-01846-s001.pdf]

Supplementary tables

Table 1: Positive cultures (monomicrobial) using the Puigvert Foundation method. Number of lost cultures and the percentage they represent within each medium at each incubation time.

**Microorganism**	**N. º of positive cultures PF method**	**N. º of false negative cultures in CRA 24 h**	**N. º of false negative cultures in CRA 48 h**	**N. º of false negative cultures in BA 24 h**	**N. º of false negative cultures in BA 48h**
Escherichia coli	106	2	1	0	0
Klebsiella pneumoniae	41	1	1	0	0
Pseudomonas aeruginosa	21	3	0	0	0
Enterococcus faecalis	14	0	0	0	0
Proteus mirabilis	22	2	0	1	1
Staphylococcus aureus	8	1	0	0	0
Enterobacter cloacae	3	0	0	0	0
Klebsiella oxytoca	4	0	0	0	0
Staphylococcus epidermidis	5	3	0	0	0
Viridans streptococci	3	2	2	1	0
Streptococcus anginosus	3	3	2	0	0
Candida parapsilosis	4	3	1	1	0
Citrobacter koseri	4	0	0	0	0
Serratia marcescens	2	0	0	0	0
Corynebacterium urealyticum	2	1	1	0	0
Enterobacter aerogenes	2	0	0	0	0
Enterococcus faecium	3	0	0	0	0
Staphylococcus hominis	2	0	0	0	0
Staphylococcus haemolyticus	1	0	0	0	0
Streptococcus agalactiae	1	0	0	0	0
Proteus vulgaris	1	0	0	0	0
Citrobacter freundii	2	0	0	0	0
Morganella morganii	2	0	0	0	0
Candida albicans	2	0	0	0	0
Stenotrophomonas maltophilia	1	1	0	1	0
Candida glabrata	1	1	1	1	0
Streptococcus sanguinis	1	1	0	0	0
Aerococcus viridans	1	0	0	0	0
Coag. neg. *Staphylococcus*	1	0	0	0	0
Proteus penneri	1	0	0	0	0
Enterobacter cancerogenus	1	0	0	0	0
Acinetobacter baumanii	1	0	0	0	0
TOTAL	266	24 (9.02%)	9 (3.38%)	5 (1.88%)	1 (0.38%)

The percentage of lost microorganisms in each case is shown relative to the total isolates of the same genus/species in parenthesis.
CRA: Chromogenic agar.
BA: Columbia-based blood agar.

Table 2: Comparison of positive cultures lost in different methods.

**Method 1 *vs* method 2**	**N. º of false negative cultures in method 1 out of total positive cultures [(a)]**	**p (McNemar)**	**Percentage of false negative cultures in method 1 compared to method 2**	**Confidence interval (95%) Z-score**	**Percentage increase of method 2 compared to method 1**	**Confidence interval (95%) Z-score**
CRA 24 h – BA 48 h	23	<0.0001	8.65 (265)	5.56 – 12.70	9.39	6.05 - 13.76
CRA 24 h – FP method	24	<0.0001	8.99 (266)	5.85 – 13.08	9.80	6.37 - 14.22
BA 24 h – BA 48 h	4	0.125	1.50 (265)	0.41 – 3.80	1.51	0.42 - 3.84
BA 24 h – FP method	5	0.0625	1.87 (266)	0.61 – 4.31	1.89	0.62 - 4.36
BA 48 h – FP method	1	1	0.38 (266)	0.009 – 2.08	0.37	0.009- 2.06
CRA 48 h – BA 48 h	8	0.0391	3.01 (265)	1.31 – 5.84	3.08	1.33 - 5.96
CRA 48 h – FP method	9	0.0039	3.37 (266)	1.55 – 6.30	3.46	1.59 - 6.47
CRA 24 h – BA 24 h	19	<0.0001	7.25 (262)	4.42 – 11.09	7.76	4.74 - 1.85
CRA 24 h – CRA 48 h	15	0.0001	5.81 (258)	3.19 – 9.40	6.12	3.47 - 9.89

Statistical calculations: p-value for the difference in proportions of positive cultures obtained with the various methods and confidence intervals for the lost proportions. Calculation of the percentage increase of method 2 compared to method 1.
